# Decoquinate liposomes: highly effective clearance of *Plasmodium* parasites causing severe malaria

**DOI:** 10.1186/s12936-022-04042-8

**Published:** 2022-01-24

**Authors:** Sumei Zeng, Hongxing Wang, Long Tao, Xiaohui Ning, Yinzhou Fan, Siting Zhao, Li Qin, Xiaoping Chen

**Affiliations:** 1 Guangzhou Bluelight Pharmaceutical Technology Co., Ltd, International Business Incubator, Guangzhou Science Park, Guangzhou, 510663 China; 2 CAS Lamvac Biotech Co. Ltd, International Business Incubator, Guangzhou Science Park, Guangzhou, 510663 China

**Keywords:** Decoquinate, Liposomes, Severe malaria, *Plasmodium*

## Abstract

**Background:**

Severe malaria caused by *Plasmodium falciparum* leads to most malaria-related deaths globally. Decoquinate (DQ) displays strong activity against multistage infection by *Plasmodium* parasites. However, the development of DQ as an oral dosage form for the treatment of malaria at the blood stage has not been successful. In this study, liposome formulations of DQ were created for intravenous (IV) injection to suppress *Plasmodium berghei*, a parasite that causes severe malaria in mice.

**Methods:**

DQ liposomes were prepared by conventional ethanol injection method with slight modifications and encapsulation efficiency evaluated by the well-established centrifugation method. Potency of the DQ liposomes against *P. falciparum* was assessed in vitro using freshly isolated human red blood cells. The efficacy of the DQ liposomes was examined in the mouse model of severe malaria.

**Results:**

The DQ liposomes were around 150 nm in size and had the encapsulation efficiency rates > 95%. The freshly prepared and lyophilized liposomes were stable after storage at − 20 °C for 6 months. The liposomes were shown to have excellent activity against *P. falciparum* in vitro with DQ IC_50_ 0.91 ± 0.05 nM for 3D7 (chloroquine sensitive strain) and DQ IC_50_ 1.33 ± 0.14 nM for Dd2 (multidrug resistant strain), which were 18- and 14-fold more potent than artemisinin, respectively. Mice did not have any signs of toxicity after receiving high dose of the liposomes (DQ 500 mg/kg per mouse) by IV injection. In the mouse model of severe malaria, the liposomes had impressive efficacy against *P. berghei* with DQ ED_50_ of 0.720 mg/kg.

**Conclusion:**

The DQ liposomes prepared in this study were stable for long term storage and safe for IV injection in mammalian animals. The newly created liposome formulations had excellent activity against *Plasmodium* infection at the blood-stage, which encourages their application in the treatment of severe malaria.

## Background

Malaria caused by *Plasmodium* parasites can be a deadly disease if not treated properly. Among the species causing human infection, *Plasmodium falciparum* is the most prevalent species in sub-Saharan Africa responsible for most malaria deaths globally. The remaining species are not typically as life-threatening as *P. falciparum.* In severe malaria, rapid control of infection is the key to keep the patient alive. Artemisinin-based combination therapy (ACT) is the first-line treatment of *P. falciparum* malaria. However, the treatment with this medication has encountered problems with the emergence of malaria recrudescence and drug resistance [1].

Treating severe malaria with intravenous or intramuscular artesunate, one of the artemisinin derivatives, has been recommended by the World Health Organization (WHO) [2]. Intravenous artesunate (AS) has typical AS half-life estimates of less than 15 min. Within about 25 min, AS is converted to its active dihydro-artemisinin (DHA) which has a slightly longer half-life of 30–60 min. Therefore, the overall average half-life ranges from 0.5 to 1.5 h [3]. The relative short half-life consequently requires frequent administration, leading to the probability of drug resistance [4]. Additionally, there has been a concern about the embryotoxicity of artesunate [5, 6].

Decoquinate (DQ), a quinolone derivative, is inexpensive and commercialized for use in controlling coccidiosis in poultry and domestic ruminants for several decades wherein it displays negligible toxicity [7]. Additionally, it has potent activity against *Plasmodium* parasites [8]. The mechanism by which it suppresses the parasites is targeting mitochondrial cytochrome *bc1* complex. DQ can suppress the development of *Plasmodium berghei* in the liver, the replication of *P. falciparum* in the blood and the generation of *P. falciparum* gametocytes which play a role in transmission [9, 10]. However, due to its exceedingly poor solubility in water and majority of organic solvents, development of DQ as a clinical drug is problematical and challenging.

To improve the solubility of DQ, Wang et al. successfully used solid dispersion technique to create its nanoparticle formulations as an oral dosage form [11, 12]. Oral route administration of DQ nanoparticles has been demonstrated to significantly enhance bioavailability and the anti-malarial activity against the liver stage *Plasmodium* [12, 13], but this approach has not been proved to be effective in suppressing *Plasmodium* parasite at the blood stage. Although the inhibition of *Plasmodium* infection at the liver stage can play a significant role for chemoprophylaxis of acute infections of *Plasmodium vivax* (as well as *Plasmodium ovale*) and for prevention of malaria relapse caused by hypnozoites [14], it does not provide a meaningful solution for *Plasmodium* infection at the blood stage, as in the case of severe malaria caused by *P. falciparum*.

Once parasites egress from the liver and enter the bloodstream, they infect the red blood cells. Patients suffering from *P. falciparum* infection at the blood stage manifest typical symptoms like shivering, high fever, headache, and anaemia. If not treated promptly, the symptoms can be worsened and as a result, the condition quickly develops into severe malaria. The patients may be unable to take oral medications if they develop clinical symptoms such as impaired consciousness, nausea, and vomiting.

Liposomes are nano- and micro-sized colloidal multilayer vesicles comprising an aqueous compartment enclosed by lipid materials and have been widely used as an ideal drug-carrier system. Liposomes have a plethora of advantages due to their similarity with cellular membranes and their ability to incorporate various substances, and their biocompatibility, enhanced drug bioavailability, sustained or controlled drug release, excellent biodegradability, and improved therapeutic index of drug [15]. Liposomes have been explored for parenteral delivery of anti-malarial drugs, such as chloroquine [16], artemisinin [17], and artesunate [18]. Therefore, liposomal creation of DQ would be a favourable alternative to solve the problem of its extremely low solubility to improve its bioavailability and provide parasiticidal therapy of severe falciparum malaria.

Currently, liposomes may be prepared by conventional methods such as thin-film hydration, reversed phase evaporation, detergent dialysis, and solvent-injection techniques. Post-processing procedures, such as sonication, high pressure extrusion and homogenization are commonly used for reducing the size of vesicles. More novel methods for preparing liposomes involve supercritical fluid technology, dual asymmetric centrifugation, crossflow filtration technology, membrane contactor technology, and others [19]. For the novel technologies, sophisticated and expensive systems are needed. These drawbacks limit the interest in the production of liposomes at a scaling-up.

The method selected for preparing liposomes depends on the physicochemical property of individual compounds. In evaluating different techniques for preparing the DQ liposomes, the ethanol injection method was chosen. The biocompatibility and safety of DQ liposomes were assessed by haemolysis assay and acute toxicity test. In vitro anti-malarial activity of liposomal DQ against *P. falciparum* (chloroquine sensitive strain 3D7 and multidrug resistant strain Dd2) was screened using the SYBR-green I-based fluorescence assay. In vivo anti-malarial efficacy was examined in the mouse model using the Kunming mice. Pharmacokinetic experiments were performed by using Sprague–Dawley rats and LC–MS/MS analysis.

## Methods

DQ was bought from Zhejiang Genebest Pharmaceutical Co., Ltd. (Zhejiang, China). Injection grade soy phosphatidylcholine (SPC) was bought from Shanghai Tywei Pharmaceutical Co., Ltd, (Shanghai, China). Egg phosphatidylcholine (EPC), hydrogenated soy phosphatidylcholine (HSPC), and cholesterol were provided by Guangzhou HanFang Pharmaceutical Co., Ltd. (Guangzhou, China). Poloxamer 188 (P188) and macrogol 15 hydroxystearate (HS15) were provided by BASF SE (Ludwigshafen, Germany). Injection grade polyethylene glycol 400 (PEG400) was bought from Jiangxi Yipusheng Pharmaceutical Co., Ltd, (Jiangxi, China). Injection grade sucrose was bought from Pfanstiehl, Inc. (Waukegan, IL, USA). All other chemical reagents were of analytical grade commercially available, and solvents and water used in analytic measurements were of HPLC grade.

### Preparation of DQ liposomes

DQ liposomes were prepared by ethanol injection method with slight modifications [20, 21]. Ingredients including DQ, phosphatidylcholine, and/or cholesterol or P188 were co-dissolved in anhydrous ethanol by heating up to 78 °C and then rapidly injected into an aqueous phase stirred at 800 rpm and comprising double distilled water containing either PEG400 or HS15.

As shown in Fig. 2, freshly prepared F5 DQ liposomes appeared to be light milk like, homogenous and had no visible particles and precipitates; their particle sizes were relatively small (133.1 ± 1.4 nm). Lyophilized powder of the liposomes was white and in a loose state while the aqueous suspension of the DQ liposomes from the lyophilized powder of freeze–dried liposomes appeared to be transparent and light-blue colored. Tangential flow filtration (TFF) system with 100 kDa pores hollow fiber membranes (mPES, Spectrum Laboratories Inc.) was used to eliminate excessive water and to concentrate liposomes at room temperature with the TFF peristaltic pump set at a rate of 80 rpm. The formulations for laboratory scale preparation of the DQ liposomes are shown in Table 1.

**Table 1:** Ingredients and proportions of each component in preparation of DQ liposome formulation

Formulation	SPC (mg)	DQ (mg)	Chol (mg)	P188 (mg)	PEG 400 (mg)	EtOH (ml)	H_2_O (ml)	Other (mg)
F1	80	20	–	–	–	15	285	–
F2	40	20	10	–	–	30	270	–
F3	80	40	20	–	65	30	270	–
F4	80	40	7	60	102	15	285	–
F5	80	20	–	160	65	15	285	–
F6	–	20	–	160	65	15	285	80 (EPC)
F7	–	20	–	160	65	15	285	80 (HSPC)
F8	80	40	–	–	–	30	270	130 (HS15)

*DQ* decoquinate, *SPC* soy phosphatidylcholine, *Chol* cholesterol, *P188* poloxamer 188, *PEG400* polyethylene glycol 400, *EtOH* anhydrous ethanol, *EPC* egg phosphatidylcholine, *HSPC* hydrogenated soy phosphatidylcholine, *HS15* macrogol 15 hydroxystearate

The drug load is based on solid ingredients used in formulations. As shown in Table 1, DQ loads of F1, F2, F5 and F8 are 20%, 28.57%, 6.15% and 33.33%, respectively. In the case of F5 liposomes according to Table 1, there is 20 mg of DQ and 325 mg of the whole liposome. For scale-up preparation, the composition and ratios of ingredients were unchanged relative to laboratory scale and the speed for agitation during injection and TFF pump rate remained almost the same (Table 2).

**Table 2:** Proportions of each component and experimental conditions in scale-up preparation of F5 and F8 liposomes

Large scale	F5 liposomes	F8 liposomes
Ingredients		
DQ (mg)	200	400
SPC (mg)	800	800
P188 (mg)	1600	–
PEG400 (mg)	6500	–
HS15 (mg)	–	1300
EtOH (ml)	150	300
H_2_O (ml)	2850	2400
Total volume (ml)	3000	3000
Stirring time post mixing (min)	5	5
Stirring speed (rpm)	1000	1000

*DQ* decoquinate, *SPC* soy phosphatidylcholine, *P188* poloxamer 188, *PEG400* polyethylene glycol 400, *HS15* macrogol 15 hydroxystearate, *EtOH* anhydrous ethanol, *TFF* tangential flow filtration

### Lyophilization and rehydration

Liposomal suspensions containing DQ were passed through a 0.22 µm filter after concentration. The suspensions were then mixed with added sucrose (sucrose: PC = 4:1, w:w) as cryoprotectant, filled in glass vials, and sealed with rubber closures. The liposome samples were frozen at − 80 °C for 12 h and lyophilized in a freeze dryer (FD-1A-50, Biocool Instrument Co., Ltd., China) at − 55 °C for 48 h.

Lyophilized power was resuspended in a 5% glucose solution for use in animal study by IV injection. Before injection, pH values were measured by a digital pH meter (PB-10, Sartorius, Germany).

### Physicochemical property analysis

Appearance of liposomes was observed by high resolution transmission electron microscopy (TEM) apparatus (Tecnai G2 FEI) at accelerating voltage of 120 kV. Liposome 100 µl was negatively stained with 2% (w/v) phosphotungstic acid solution and placed on a 400-mesh carbon-coated copper grid. The sample was left to air dry before analysis. Average hydrodynamic diameter, polydispersity index (PDI), and zeta potential of DQ liposomes were determined by Malvern Zetasizer Nano ZSE (Malvern Instruments Ltd., UK). The concentrated liposomes before lyophilization and the hydrated liposomes after lyophilization were measured in triplicate after dilution with sterile water. Average diameter of particle sizes and PDI value were obtained at an angle of 175° in 1 cm diameter cells at 25 °C. Zeta potential was measured by determining electrophoretic mobility under an electric field and expressed as an average of 10–30 measurements.

### Determination of DQ content and encapsulation efficiency (EE %)

DQ content of liposomes was estimated by a high-performance liquid chromatography (HPLC) system (Agilent 1260, USA). Diamonsil C18 column (250 × 4.6 mm, 5 µm; Beijing Dikma Technologies, Inc. China) was used to separate DQ from other components in the sample. The mobile phase consisted of 80% ethanol and 20% water (0.1% formic acid contained in water and ethanol, respectively).

Liposomal DQ was determined by HPLC for all efficacy, toxicity, and pharmacokinetic experiments to calculate concentrations of DQ in cell culture or dosages of DQ in animals. For instance, in the efficacy experiment, a mouse weighing 20 g dosed at 10 mg/kg of DQ would receive 200 μg of DQ suspended in 100 μl volume, according to the HPLC quantitation. In the case of F5 liposomes, 3.25 mg of the liposomes would be injected via the tail vein. Similarly, for the pharmacokinetic experiments in rats, 10 mg/kg of DQ was given to a rat of 200 g, which would be 2 mg of F5 liposomal DQ or 32.5 mg of the whole liposomes.

For encapsulation efficiency, 1 ml liposome suspensions were added to each tube and centrifuged at 5000 rpm for 10 min at 25 °C. The supernatant was analysed and DQ measured by HPLC. Encapsulation efficiency was calculated by dividing the amount of DQ in the supernatant by the total amount of DQ in the sample before the centrifugation.

### Stability of the DQ liposomes

The stability of DQ liposomes under aqueous and powder conditions was checked after storage. The liquid form of freshly prepared DQ liposomes was stored at 4 °C for 5 days, and assessed for changes in liposome size, PDI, zeta potential, and EE%. The same assessment was performed for the lyophilized powder of the DQ liposomes after storage at − 20 °C over a period of 6 months and rehydration at specified time intervals (0, 2, and 6 months of storage).

### Haemolysis assay

To examine if liposomes induce cell-wall lysis [22], the haemolytic effects of the DQ liposomes were examined by using the method of Vijayakumar et al. [23]. F5 and F8 liposomes were used for the assay. Briefly, 5 ml of fresh blood were collected from a healthy volunteer and centrifuged at 3000 rpm for 10 min. Plasma layer was removed and erythrocytes washed three times with normal saline (NS). The washed erythrocytes were diluted up to 50 ml using NS. Then, 0.3 ml, each from F5 and F8 at two different concentrations (equivalent to 1 mg/ml and 0.1 mg/ml of DQ) was added separately to the tubes containing 2.5 ml of erythrocyte suspension and 2.2 ml NS. Equal volume of NS mixed with 2.5 ml erythrocyte suspension was used as negative control, while 2.5 ml of erythrocyte suspension diluted with 2.5 ml of double distilled deionized water was set as positive control. After the incubation at 37 °C for 3 h, the samples were centrifuged at 3000 rpm for 10 min. Then1 ml of supernatant was transferred to a new tube and 100 µl of 10% triton X-100 added. Lastly, the released haemoglobin was measured spectrophotometrically at 540 nm to calculate the percent haemolysis as:

$$\% {\text{ Haemolysis}} = {{\left( {{\text{A}}_{{\text{s}}} - {\text{A}}_{{\text{n}}} } \right)} \mathord{\left/ {\vphantom {{\left( {{\text{A}}_{{\text{s}}} - {\text{A}}_{{\text{n}}} } \right)} {\left( {{\text{A}}_{{\text{p}}} - {\text{A}}_{{\text{n}}} } \right)}}} \right. \kern-\nulldelimiterspace} {\left( {{\text{A}}_{{\text{p}}} - {\text{A}}_{{\text{n}}} } \right)}} \times 100\% ,$$

where A_s_ is the absorbance of treatment groups, while A_n_ and A_p_ belongs to negative and positive controls, respectively.

### Acute toxicity

In vivo acute toxicity was assessed by the method of maximal feasible dose (MFD) according to ICH guidelines [Guidance on nonclinical safety studies for the conduct of human clinical trials and marketing authorization for pharmaceuticals M3 (R2)]. The Kunming mice (6–8 weeks old, weighing around 30 g) were divided into two groups (n = 8, 4 males and 4 females). Mice were given F5 liposomes with a single high dose of DQ 500 mg/kg body weight via the tail vein in the treatment group, and the liposome vehicle was given to the control group. The mice were observed for 1 h after the liposome injection and once a day for the next 13 days. Body weights were recorded for the surviving mice and visual observation included grooming, writhing, alertness, convulsions, salivation, vomiting, and urination.

### In vitro anti-malarial activity

In the in vitro anti-malarial bioassay, strains of *P. falciparum* (chloroquine sensitive 3D7 and multidrug resistant Dd2) were kept in continuous culture in human erythrocytes in RPMI 1640 medium supplemented with 0.5% Albumax I/II and 0.37 nM hypoxanthine according to the method previously described by Trager and Jensen [24]. Synchronization of parasites was conducted using sorbitol treatment [25] and parasitaemia was microscopically determined in Giemsa-stained smears.

In vitro anti-malarial activity assay was performed as previously described [26]. Chloroquine and artemisinin were used as standard anti-malarial drugs, and equivalent free DQ used as a control and dissolved in dimethylsulfoxide (DMSO). Liposomes were diluted into a series of 10 µl different DQ concentrations for the in vitro assay. Each sample (10 µl) was mixed with 90 µl culture medium containing *P. falciparum* (0.5% parasitaemia and 2% haematocrit) and placed in 96-well microplates, which were incubated for 72 h with 5% CO_2_ at 37 °C. Lysis buffer (100 µl) containing 0.2 µl SYBR- Green I was then added to each well of the plate and incubated for additional 1.5 h at 37 °C. Fluorescence signal was detected at 488 nm (excitation) and 520 nm (emission) in a microplate reader (Synerfy H1, Bio Tek, USA). The intensity of fluorescence generated by the binding of SYBR-Green I to *Plasmodium* DNA is relative to the growth of parasites [27]. IC_50_ values were derived from dose–response curves evaluated with software GraphPad.

### In vivo anti-malarial efficacy

In the preliminary experiment, mice (n = 5) in control and experimental groups were inoculated intraperitoneally with infected blood containing 1 × 10^7^ *Plasmodium berghei* (strain ANKA) and the blood infection monitored. At day 4 (96 h), parasitaemia rose to about 30% (22.8–38.3%) and DQ at 6.4 mg/kg formulated in lipid vesicles was intravenously given to mice in experimental group. At day 6 (144 h), second dose of the formulated DQ was administered to experimental mice. At day 8, all mice receiving DQ survived and their parasitaemia fell to nearly zero level (data not shown). At day 8, however, of the 5 control mice, three died and two were seriously ill with parasitaemia rates as high as 60%. At the same day (day 8), the two control mice with severe malaria received the formulated DQ (6.4 mg/kg). They were then rescued and completely recovered 2 days later after the second DQ dose.

**Fig. 1:**
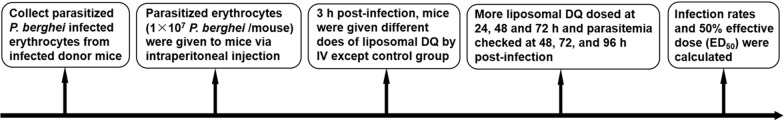
Diagrammatic sketch of efficacy evaluation of DQ liposomes

Based on the preliminary data, experiments were designed for dose response of liposomal DQ by following the protocol (Peters 4-day test) as previously described [28–30]. Mice were given F5 liposomes 3 h post inoculation and another three doses at 24 h, 48 h and 72 h afterwards (Fig. 1). Although the treatment 3 h after infection does not exactly reflect human situation of severe malaria, there is a consensus on the similarities between findings in rodent models and malaria and the role of animal models for research on severe malaria [31]. Anti-malarial efficacy experiments in animals were conducted in compliance with the Chinese State Council’s Laboratory Animal Management Regulations (Revised March 1, 2017). The Kunming mice (6–8 weeks old, weighing around 30 g) were kept in their crates for at least 7 days after arrival. Each mouse was housed in a cage at a temperature of 22 ± 4 °C with a relative humidity of 40–70%, and the room kept in a 12 h light/12 h dark cycle. Ten mice (n = 10) were assigned for each group. Erythrocytes (PE) parasitized with 1 × 10^7^ *P. berghei* from donor mice was injected intraperitoneally to each naive mouse on day 0. Three hours post infection, F5 liposomes with 6 different DQ doses at 0.04, 0.12, 0.37, 1.11, 3.33, and 10.0 mg/kg body weight were injected intravenously to the infected mice assigned in each dosage group through the tail vein. Mice in control group were treated with normal saline. Administration of the DQ liposomes was repeated 24, 48, and 72 h post-infection and peripheral blood smears were prepared by making a thin film with a drop of blood taken from the tail vein of each animal at 48, 72 and 96 h post-infection. The blood smears were fixed in methanol and stained with 10% Giemsa for 20 min. Parasitaemia was determined under microscope with oil immersion by counting the number of infected RBCs per 1000 RBCs. The 50% effective dose (ED_50_) was estimated based on the parasitaemia at 96 h post-infection. The blood smears were examined every 3 days for 30 days after the treatment was completed.

### Pharmacokinetics

F5 liposomes were intravenously given to Sprague–Dawley rats (weight 200–250 g) with DQ at a single dose of 10 mg/kg body weight. The liposomes were diluted with aqueous solution into concentration of DQ at 3 μg/μl. For a 200 g rat, 2 mg of DQ corresponding to 32.52 mg of the liposomes would be administered. The blood was collected from the retro-orbital vein and the rats monitored for any adverse effects within 2 days after blood collection. The time points for the blood collection were at 0.17, 0.5, 1, 2, 3, 4, 6, 8, 12, 24, and 48 h post-injection. The whole blood samples were treated by protein precipitation method. A solution of 20 μl ethanol and 100 μl blood sample were added to a 1.5 ml tube and mixed by vortex for 3 min. Then 400 μl of protein precipitation reagent (ethanol:acetonitrile = 1:1) containing internal standard was added and mixed by vortex for 5 min. The tubes were covered and left at room temperature overnight. The samples were mixed by vortex for 5 min and then centrifuged at 16000×*g* for 30 min at 4 °C. The supernatant (100 μl) from each sample was transferred to 96 well plate and analysed by LC–MS/MS (Agilent Technologies 647 0 Triple Quad LC/MS). The pharmacokinetic (PK) analysis consisted of C8 column (2.1 × 50 mm, 3.5 µm; Agilent ZORBAX Eclipse Plus) with mobile phase comprising 90% methanol (containing 0.1% formic acid) and 10% water (containing 0.1% formic acid) at flow rate of 300 µl/min under the temperatures at 30 °C and 15 °C for the column and the injector, respectively. The PK data were obtained from LC–MS/MS measurements of DQ in the whole blood by compartment analysis with the Drug and Statistics (DAS) program.

## Results

### Characterization of the DQ liposomes

**Fig. 2:**
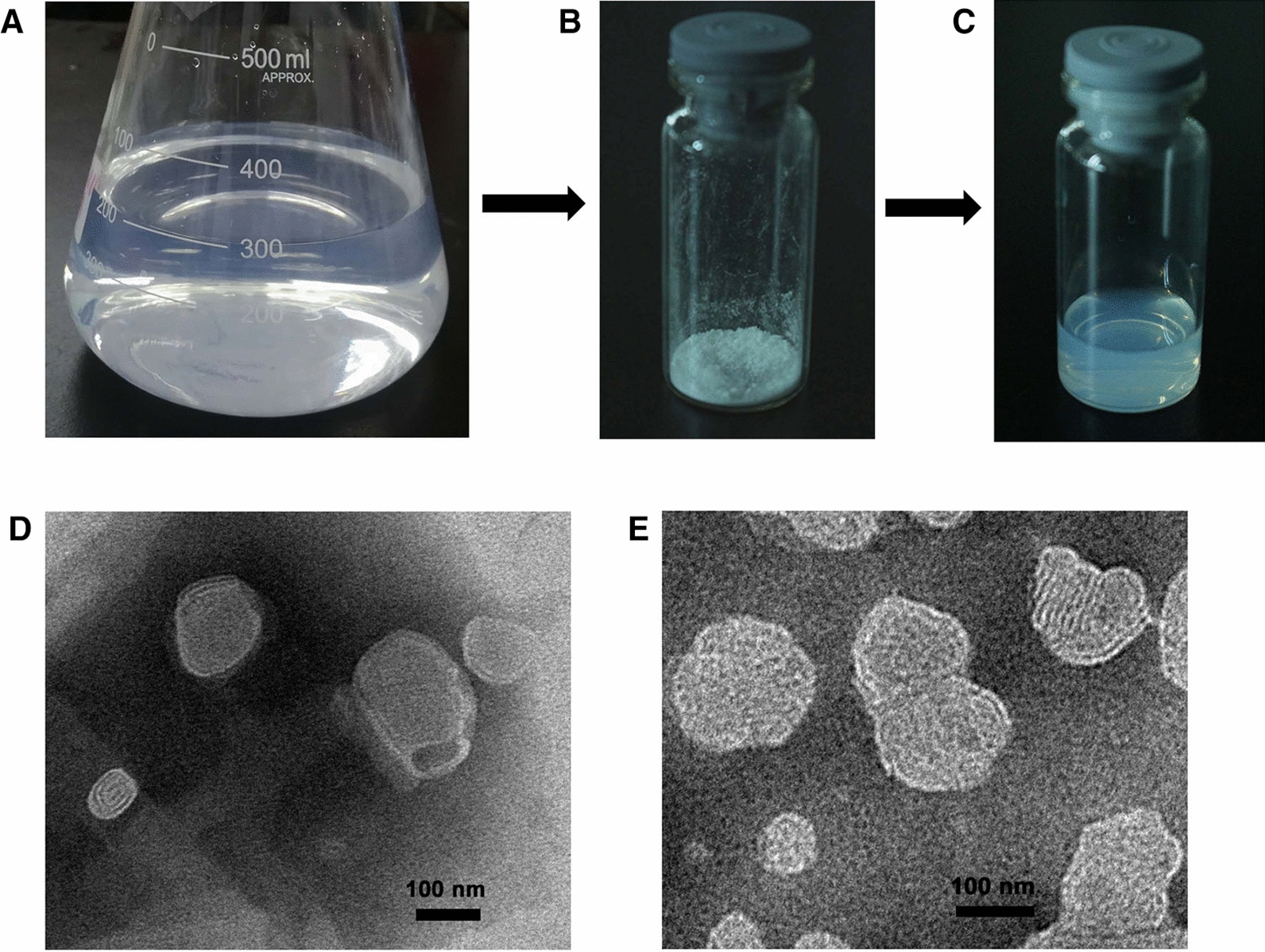
Appearances of F5 DQ liposomes. **A** Fresh suspension of the DQ liposomes prepared by the conventional ethanol injection method; **B** powder of the DQ liposomes after concentration and lyophilization in a freeze dryer at − 55 °C for 48 h; **C** suspension of the DQ liposomes from the lyophilized powder in 5% glucose solution; **D** TEM image of freshly prepare DQ liposomes; **E** TEM image of rehydrated DQ liposomes

**Fig. 3:**
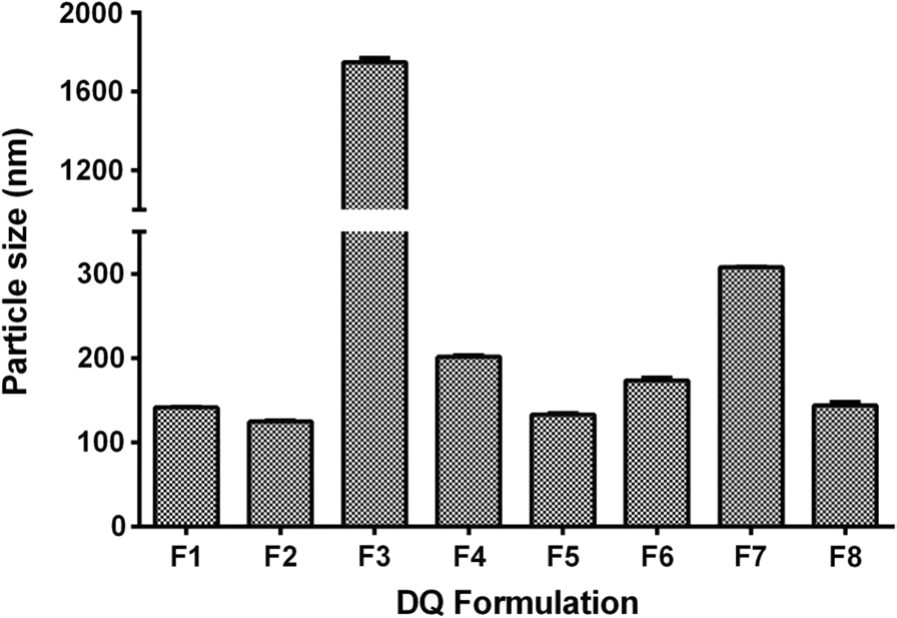
Average sizes of the DQ liposomes with different formulations. DQ liposomes were prepared according to the conventional ethanol injection method with some modifications, and 1 ml of freshly prepared DQ liposome solution was used to measure particle size by Malvern Zetasizer Nano ZSE at an angle of 175° in 1 cm diameter cells at 25 °C

The aqueous suspension of F5 liposomes appeared to be transparent and light-blue colored. Shown in Fig. 2 were freshly prepared liposomes, lyophilized powder, and rehydration from freeze–dried liposomes. The micro-structures of freshly prepared liposomes (Fig. 2D) and rehydrated liposomes (Fig. 2E) were obtained from TEM analysis and shown to be regular spherical shape with a nanometric size. The particle sizes of the liposomes were displayed in Fig. 3 and those with the average particle sizes below 150 nm were selected for further evaluation (see Table 3).

**Table 3:** Physicochemical property evaluations of DQ liposomes in 0 and 5 days after preparation (n = 3)

Measurements	Days	Samples			
		F1	F2	F5	F8
PS (nm)	0	141.5 ± 0.3	124.7 ± 1.3	133.1 ± 1.4	144.1 ± 4.0
	5	146.9 ± 2.6	153.3 ± 1.1	149.8 ± 0.4	153.6 ± 0.6
PDI	0	0.266	0.200	0.269	0.213
	5	0.259	0.194	0.271	0.234
Zeta potential (mV)	0	0.90 ± 0.17	2.21 ± 0.62	− 17.4 ± 0.59	− 6.59 ± 0.33
	5	1.11 ± 0.11	5.31 ± 0.34	− 19.9 ± 0.76	− 9.76 ± 0.47
EE (%)	0	98.8 ± 2.0	96.3 ± 2.2	98.7 ± 1.8	99.2 ± 3.0
	5	99.3 ± 2.4	95.9 ± 1.6	99.6 ± 1.6	99.8 ± 2.4

*PS* particle size, *PDI* polydispersity index, *EE* encapsulation efficiency

The DQ liposomes shown in Table 3 had excellent encapsulation rates whether freshly prepared or 5 days after preparation, all above 95%. The particle size and the zeta potential were not significantly changed, indicating the relatively stable property of the liposomes analysed. Average size of F1 DQ liposomes was increased from 141.5 to 205.7 nm after F1 was concentrated, and F2 DQ liposomes agglomerated during the process of concentration. They were not lyophilized for further stability evaluation.

**Fig. 4:**
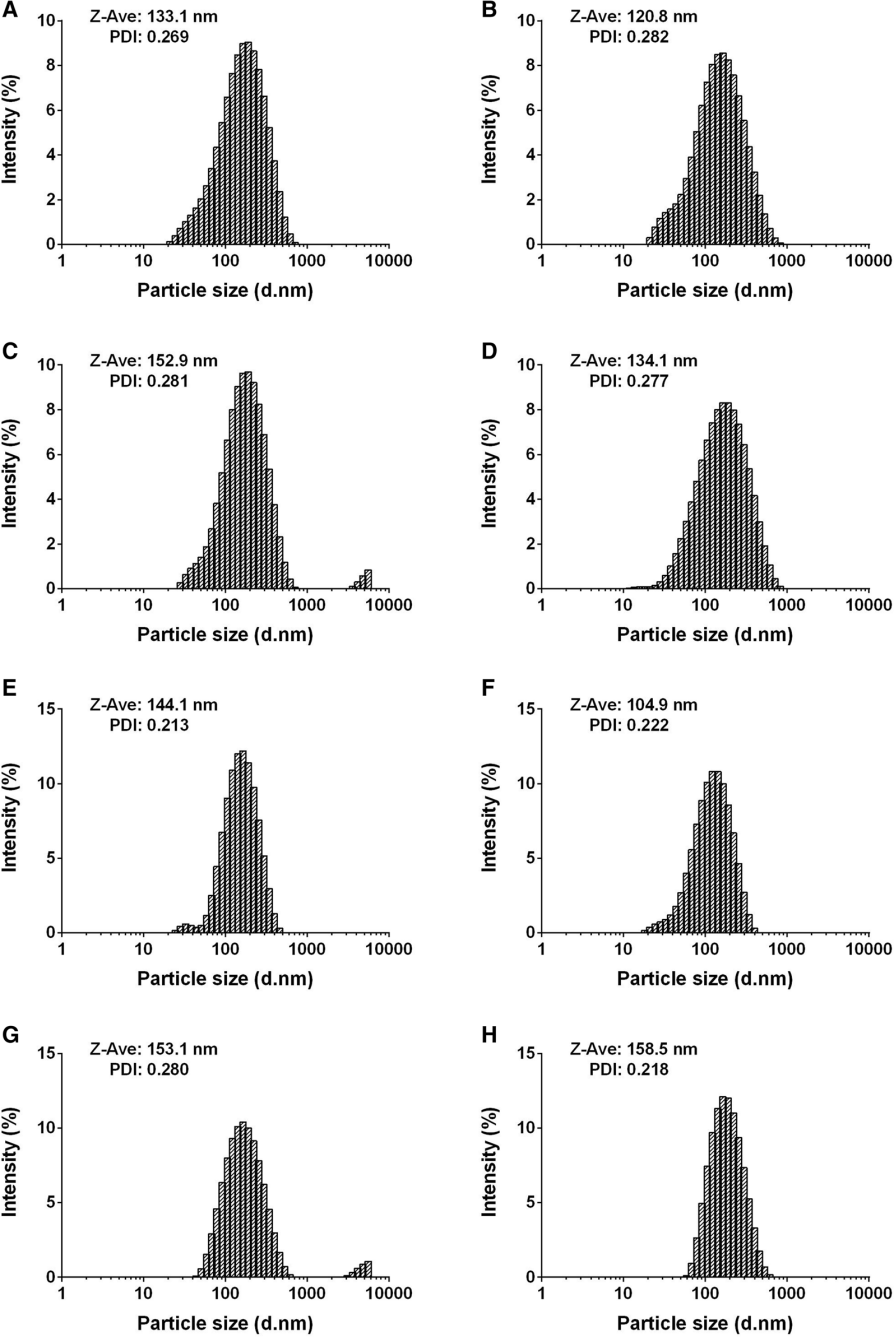
Size distribution of the DQ liposomes. **A** Freshly prepared F5 liposomes; **B** concentrated F5 liposomes; **C** hydrated F5 liposomes from lyophilized powder; **D** F5 liposomes prepared in large scale; **E** freshly prepared F8 liposomes; **F** concentrated F8 liposomes; **G** hydrated F8 liposomes from lyophilized powder; **H** F8 liposomes prepared in large scale

The particle sizes of the DQ liposomes (F5 and F8) whether freshly prepared, concentrated, lyophilized, or hydrated or prepared on large scale were not dramatically different (Fig. 4); all polydispersity index (PDI) remained below 0.3, indicating a stable particle size distribution during liposomal preparation and their quality with respect to the size distribution [32, 33].

**Fig. 5:**
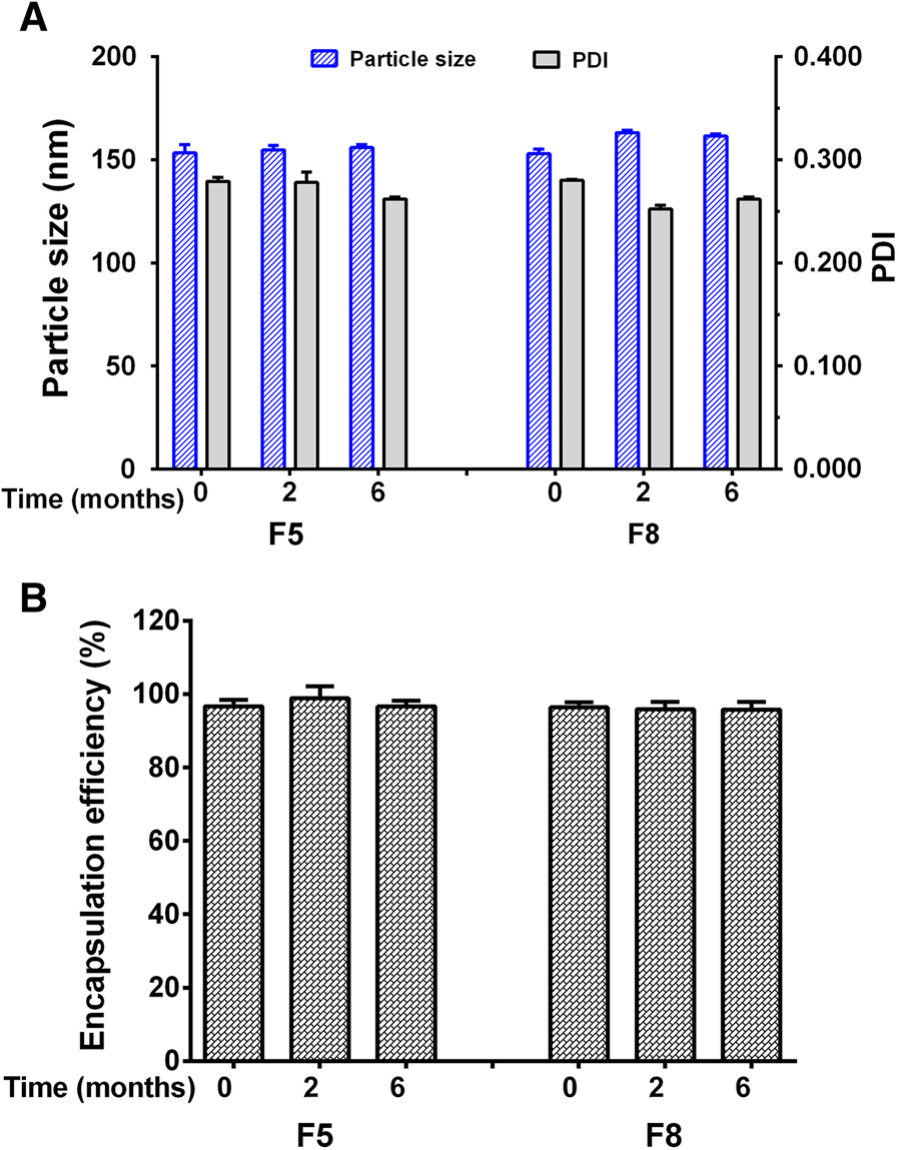
The stability of lyophilized powder of F5 and F8 DQ liposomes after storage at − 20 °C for 2 and 6 months, respectively. **A** Particle sizes and PDI; **B** encapsulation efficiency (%)

The effects of storage at − 20 °C for 6 months on the stability of F5 and F8 lyophilized powder were also examined. As shown in Fig. 5A, periodic measurements of particle size, PDI, zeta potential, and encapsulation indicated that the particle size remained in the range of 150–165 nm, and PDI were less than 0.3. Additionally, the encapsulation rates of DQ liposomes were found to be 95.8% for F5 and 96.7% for F8 after storage at − 20 °C for 6 months (Fig. 5B), indicating that the liposome structure remained intact, and no drug leakage from the liposomes occurred under the storage conditions. In addition, the pH values of lyophilized DQ liposomes were 7.32 for F5 and 7.06 for F8 when resuspended in 5% glucose solution. In animal experiments, therefore, F5 liposomes were selected.

### Haemolytic potential and toxicity of liposomes

The percent haemolysis of F5 liposomes was 0.73% and 0.03% for high (1 mg/ml) and low concentrations of DQ (0.1 mg/ml), respectively whereas the haemolysis rates of F8 liposomes were 2.03% and 0.35% for high and low concentrations of DQ, respectively. All haemolysis values are below 5%, which is a standard permissible for clinical use [34]. All mice receiving the two DQ liposomes appeared to be normal and survived to the end of the observation period.

For acute toxicity experiments, mice were given F5 liposomes at a dose of DQ 500 mg/kg by IV injection. No signs of toxicity were seen. Body weights of mice were basically not affected. The exact maximum tolerable DQ dose of F5 liposomes was not estimated because a much higher safe dose could be given and it would be much greater than 500 mg/kg, which was 50 times or more that of the effective dose for blood stage suppression of *P. falciparum*.

### Anti-malarial efficacy of liposomes in vitro and in vivo

In vitro assay was conducted to examine growth inhibition of *Plasmodium* parasites by liposomal DQ in cell culture and the half maximal inhibitory concentrations (IC_50_) based on dose response curves were determined (Table 4). The IC_50_ values of free DQ and F5 liposomal DQ for *P. falciparum* were compared to those of chloroquine and artemisinin, indicating its potent activity against *P. falciparum* associated with severe malaria. The data showed that the IC_50_ of F5 liposomal DQ for chloroquine sensitive *P. falciparum* 3D7 was 0.91 nM, which were respectively 26- and 18-fold lower than those of chloroquine and artemisinin. The IC_50_ of F5 liposomal DQ against multidrug resistant *P. falciparum* Dd2 was 1.33 nM, which was 13-fold lower than that of artemisinin (IC_50_ 18.53 nM). In addition to using saline as a control, the liposomes (F5 with no DQ and F8 with no DQ) were also assessed and shown to have no inhibitory effects on *P. falciparum* (Table 4).

**Table 4:** The inhibitory effects of liposomes on the growth of *Plasmodium falciparum* parasites in infected human red blood cells (iRBC), expressed by the half maximal inhibition concentration (IC50)

Preparations	*P. falciparum* 3D7		*P. falciparum* Dd2	
	Growth (n = 5)	Inhibition (n = 5)	Growth (n = 5)	Inhibition (n = 5)
	%	IC50 mean ± SD (nM)	%	IC50 mean ± SD (nM)
Liposomal DQ				
F1		2.47 ± 0.23		–
F2		2.11 ± 0.52		–
F5		0.91 ± 0.05		1.33 ± 0.14
F8		1.67 ± 0.04		1.12 ± 0.11
DQ in DMSO		1.63 ± 0.13		1.57 ± 0.49
Chloroquine		23.75 ± 0.06		184.8 ± 16.24
Artemisinin		16.44 ± 5.80		18.53 ± 0.50
Saline	100 ± 0.00		100 ± 0.00	
Liposomes				
F5 (no DQ)	100.46 ± 4.66		95.81 ± 4.95	
F8 (no DQ)	101.46 ± 4.78		99.22 ± 4.17	

Each preparation was described in the method section

Means of results are calculated from three separate experiments run on different days

*DQ* decoquinate, *DMSO* dimethylsulfoxide

**Fig. 6:**
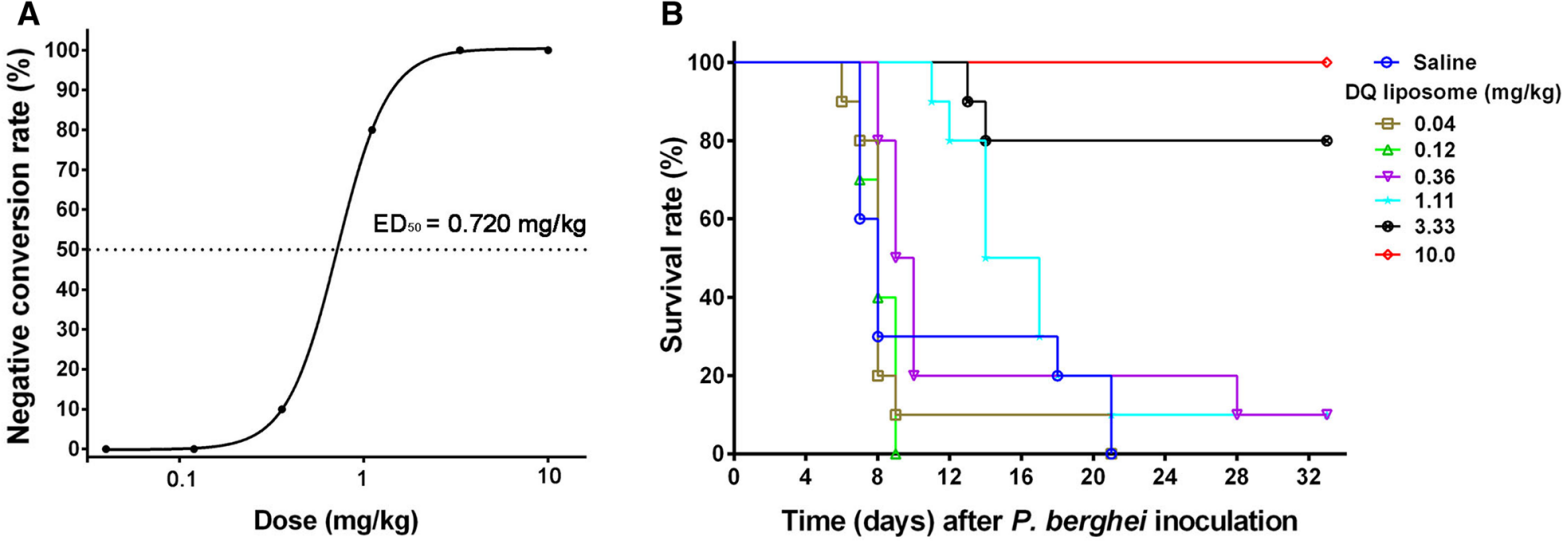
Activity of F5 liposomal DQ against *P. berghei* infection in the mouse model of severe malaria. F5 liposomes with different doses of DQ were given to mice assigned in different dosage groups at 3 h, 24 h, 48 h and 72 h post infection. Samples were prepared with blood drawn from mice 96 h post infection of *P. berghei* parasites. **A** Parasitaemias were calculated by counting cells under microscopic view of blood smears at ×100 magnification under oil immersion. Dose–response curves of F5 liposomal DQ versus suppression of parasitaemia 96 h post infection; **B** survival rates of mice infected by *Plasmodium* parasites and treated with different doses of F5 liposomal DQ

F5 liposomes were selected for in vivo test of efficacy because the physicochemical property and in vitro anti-malarial activity of F5 were better than those of other liposomes evaluated (Table 4). As compared to the saline group, all mice treated with F5 liposomes showed reduction in parasitaemia 96 h after infection. Almost no parasites could be seen in dosage groups of DQ at 1.11, 3.33, and 10.0 mg/kg/day. ED_50_ is determined as the dose in mg/kg that reduces parasitaemia by 50% at 96 h post infection (Fig. 6A). As shown in Fig. 6A, the DQ ED_50_ value of F5 liposomes was 0.720 mg/kg in mice infected with *P. berghei*. Survival curves (Fig. 6B) showed that all untreated mice succumbed to death by day 21 post-infection. After 33 days infection, mice treated with F5 liposomes at DQ 0.36, 1.11, 3.33, and 10.0 mg/kg/day had survival rates of 10%, 10%, 80%, and 100%, respectively. For mice treated with doses at DQ 3.33 and 10.0 mg/kg/day, the parasitaemia was completely inhibited. However, recrudescence occurred in mice receiving DQ 3.33 mg/kg/day, and 2 out of 10 mice became infected by day 13 and 14 post-infection and succumbed to malaria by day 32 (Fig. 6B). However, there was no recrudescence in mice dosed at 10 mg/kg/day.

### Pharmacokinetics of F5 liposomal DQ

**Fig. 7:**
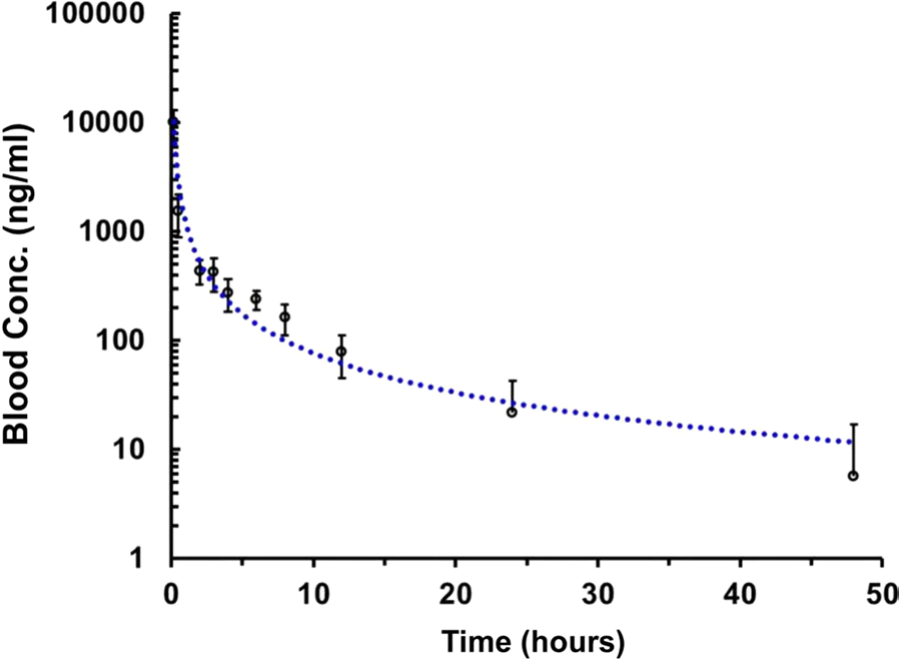
Blood concentrations of DQ after administration of F5 liposomes to Sprague–Dawley rats by IV injection at the given dose of DQ (10 mg/kg) and presented as mean ± SD (n = 4). Blood samples were collected at time points shown in the figure and blood concentrations of DQ measured by LC/MS/MS

F5 liposomes at a dose of DQ 10 mg/kg body weight were intravenously given to Sprague–Dawley rats. The blood DQ concentration–time curve is plotted in Fig. 7. Considerable reduction in circulating liposomes was observed 30 min after administration. DQ concentration in the blood dropped substantially from 10,020.22 ± 2888.15 ng/ml at 10 min to 1535.37 ± 650.31 ng/ml at 30 min (Table 5). The blood concentration was 21.70 ± 21.07 ng/ml 24 h after the liposome administration, which could still be highly effective in eliminating *Plasmodium* parasites because there has been a recent report that the minimum inhibitory concentration (MIC) of DQ for *Plasmodium* parasites is 5.12 ng/ml [35]. The effective concentration of drug in the blood must exceed the MIC to maintain the ability of animals to kill parasites [36, 37]. Thus, there could be the effective growth inhibition of parasites within 48 h after treatment.

**Table 5:** Blood concentration of DQ after intravenous administration of F5 liposome to Sprague–Dawley rats at dose of the 10 mg/kg (n = 4)

Time (h)	Blood concentration (ng/ml)	
	Mean	SD
0.167	10,020.22	2888.15
0.5	1535.37	650.31
2	435.67	108.03
3	422.77	143.24
4	274.49	91.04
6	237.49	46.44
8	162.53	51.36
12	78.24	32.75
24	21.69	21.07
48	5.72	11.24

However, the effective level of DQ concentration in the blood may not be maintained after 48 h of IV dose although the amount of DQ could be distributed still high in the peripheral tissues or organs such as the liver [11]. The PK profiles including maximum concentration (C_max_), time to maximum concentration (T_max_), half-life (t_1/2_), area under the curve (AUC), mean clearance rate (CL/F), and mean residence time (MRT) are presented in Table 6. An elimination half-life of DQ was 4.32 h and a plasma clearance of DQ was 1.04 l/kg/h for F5 liposomes.

**Table 6:** Pharmacokinetic evaluations of DQ liposomes at the dose of 10 mg/kg (n = 4) administered to Sprague–Dawley rats by intravenous injection (IV)

Parameters	Unit	Mean	SD
AUC_0−t_	µg/l	9733.08	1418.47
MRT_0−t_	h	4.53	0.77
t_1/2_	h	4.32	0.88
Vz	l/kg	6.60	2.50
CL	l/h/kg	1.04	0.16
C_max_	µg/l	10,147.02	3116

*AUC* area under the curve, *MRT* mean residence time, *t*_*1/2*_ elimination half-life, *Vz* volume of distribution, *CL* plasma clearance, *C*_*max*_ maximal concentration

## Discussion

The way to improve the solubility of a compound without abolishing or reducing its bioactivity is usually to make it into a salt to increase its hydrophilicity or through formulation methods to overcome its insolubility problem. Modifying chemical structure can play a role in improving the solubility but can also change the bioactivity. Recently, Beteck et al. generated a variety of DQ derivatives among which the carbamate derivatives of DQ had enhanced solubility and bioavailability while still retaining high selectivity for malaria parasite [38]. Such derivatives can be easily prepared at low cost.

Alternatively, the formulation technique has been applied to improve the solubility of a compound without changing the structure. Nanoparticle formulations of DQ had been prepared by the approach of co-solvency and high-pressure homogenization [11, 13]. The nanoparticles significantly improve the bioavailability as well as the efficacy. However, most drug particles gradually sediment into the bottom of the test tube when suspended in aqueous solution, which is not ideal for IV injection or for intranasal, intra-rectal and inhalation of anti-malarial drugs. Thus, the nanoparticle formulations were only assessed in mice by oral route (intragastric administration in mice). Although it showed excellent anti-malarial efficacy at the liver stage (sporozoite inoculation), many attempts to assess its activity against the blood stage infection failed. Intragastric dose of DQ leads to the high drug concentration in the liver or fat-rich organs and tissues [11] but may also suffer from the first pass phenomenon. It can play a significant role in chemoprophylaxis for *Plasmodium* infection at the liver stage but not for suppressing the infection at the blood stage. Li et al. created DQ microparticle emulsion made of oil suspended formulation to monitor the liver stage infection of *Plasmodium*. The formulation was given intramuscularly and highly effective in long-term prophylaxis [34, 35].

In this study, the DQ nanoliposomes were generated by ethanol injection technique. The nanoliposomes have similar average particle sizes to those of non-liposome nanoformulations [13], but have no precipitation when suspended in aqueous phase. The homogeneous feature of the liposomes provides a practically feasible drug delivery carrier system for parenteral route. Intravenous injection of the liposomes can quickly reach the therapeutic level of DQ in the plasma. Liposomes containing DQ are lipophilic and biocompatible, which makes it easier to be adsorbed, to enter red blood cells and to kill the blood stage *Plasmodium* parasites.

The ethanol injection technique is selectively useful for certain chemical molecules to form small liposomes [21]. In the present study, it has been proved to be suitable for packing DQ into nanoliposomes and the reproducibility of one-step-based process. However, this technique requires the use of a large volume of aqueous phase in the preparation and the elimination of ethanol post the formation. The TFF system was applied to concentrate DQ liposomes by removing most of the water and ethanol and the lyophilization system used to eliminate the rest of the water and ethanol. Lyophilization seemed to be a safe method to keep the liposomes intact and to prevent hydrolysis of phospholipids from the liposomes. In practice, the whole preparation procedures can be assembled into a novel one-step in-line liposome production processing system. On the assembled production line, liposomal suspensions containing DQ were passed through a 0.22 µm filter after concentration. The final product of liposomes is believed to be safe for IV injection. Other techniques (also tried in this laboratory) such as thin-film hydration method in which, ultrasonic technique and removal procedure of chloroform and methanol are required, increase the cost to scale up the liposome production for commercialization [39].

All inactive ingredients for the DQ liposomes have been approved by the FDA for human use as intravenous administration. Phospholipids are excipient with excellent biocompatibility and especial amphiphilicity in drug formulations [40]. Cholesterol is a common component of liposome formulation and increases the stability of liposomes. Also used in high proportion in F5 liposomes was poloxamer 188 (P188). It consists of a high portion of hydrophilic chains of polyoxyethylene which may provide steric protection to a bilayer surface [41] and stabilize liposomes in biological fluid. Polyethylene glycol (PEG) has been used for enhancing the solubility of insoluble drugs [42] and for minimizing adverse drug toxicity [43]. It has been postulated that PEG density determines its structure at the liposome surface [44]. HS15, containing 30% free PEG, is a nonionic surfactant and emulsifying agent, which is recently applied in the formation of a stable drug-delivery system [45].

During lyophilization procedure, sucrose was selected for protection of DQ liposomes based on the “water replacement” hypothesis [46, 47]. Although liposome size grew slightly, the liposomes remained relatively stable for 6 months after storage at − 20 °C. There was a slight increase in PDI of the liposomes (Fig. 4C) which could be due to insufficient aqueous suspension after lyophilization. The particle size distribution and PDI of lipid-based nanocarriers are important physical characteristics to be considered when creating pharmaceutical-grade products [32]. However, after all procedures including preparation, concentration, lyophilization, storage and reconstitution, the liposomes remained well-distributed and had particle size < 200 nm and encapsulation efficiency > 80%, which was within the quality control range of intravenous liposomes. This is well constituted, remarkably stable, and high quality of liposome product.

According to the standard of haemolysis limit that preserves the integrity and functionality of erythrocytes as described in the ASTM F756-08 [34], the haemolytic effects of F5 liposomes at both high and low DQ concentrations were within 2%, which was within the limit for clinic application. Furthermore, it is inferred that the anti-malarial effect of the liposomes towards infected RBCs was independent of haemolysis [48]. In the acute toxicity evaluation, intravenous injection of F5 liposomes at high dose (DQ 500 mg/kg) was found to be safe, the dose 50 times that of the efficacious level of anti-malarial activity.

Liposomal DQ of F5 displayed single digit nanomolar activity against blood stage parasites of *P. falciparum* 3D7 in vitro, which was in accord with previously published data [9]. F5 liposomes were also shown to effectively inhibit the multidrug-resistant *P. falciparum* strain Dd2. Since chloroquine and its derivatives were a first line anti-malarial drug in past several decades, the parasites, especially *P. falciparum*, had become drug resistant and there had been a global resurgence in malaria [49]. Although artemisinin is one of the most effective anti-malarial drugs in the blood stage as it rapidly reduces parasites load and quickly resolves clinical symptoms [50], recrudescence in late stage seems to be a problem.

In animal study, F5 liposomes examined at a relatively low dose of DQ (10 mg/kg) induced complete parasitaemia clearance without recrudescence in *P. berghei*-infected mice. Although high dose is safe, low dose for effective treatment will have an advantage in clinic application. Thus, DQ encapsulated in liposomes displays superior efficacy to currently available intravenous anti-malarials [2] and offers a promising approach by IV injection to tackle the burden of severe malaria.

## Conclusion

The DQ liposomes were highly effective in inducing complete parasitaemia clearance in *P. berghei*-infected mice without recrudescence and at relatively low doses. The liposomes were very stable and safe for IV injection. In addition, the process for preparing liposomes described here is straightforward and can be readily scaled up; it therefore appears suitable for industrial scale production. The liposome product has potential of becoming a remedy in the treatment of severe malaria, especially for the emergent use in alleviating acute lethal complications and perhaps saving lives.

## Data Availability

The datasets used and/or analysed during the current study are available from the corresponding author on reasonable request.

## Abbreviations

DQ: Decoquinate
IC_50_: 50% inhibiting concentration
ED_50_: 50% effective dose
SPC: Soy phosphatidylcholine
EPC: Egg phosphatidylcholine
HSPC: Hydrogenated soy phosphatidylcholine
P188: Poloxamer 188
HS15: Macrogol 15 hydroxystearate
PEG400: Polyethylene glycol 400
HPLC: High performance liquid chromatography
TFF: Tangential flow filtration
PDI: Polydispersity index
EE%: Encapsulation efficiency
NS: Normal saline
MFD: Maximal feasible dose
DMSO: Dimethylsulfoxide
PK: Pharmacokinetics
C_max_: Maximum concentration
T_max_: Time to maximum concentration
t_1/2_: Half-life
AUC: Area under the curve
CL/F: Mean clearance rate
MRT: Mean residence time
